# The effect of heating method on the gel structures and properties of surimi prepared from Bombay duck (*Harpadon nehereus*)

**DOI:** 10.3389/fnut.2022.1060188

**Published:** 2022-11-24

**Authors:** Mingao Li, Jing Yang, Hongli Bao, Yi Chen, Yuanpei Gao, Shanggui Deng

**Affiliations:** Key Laboratory of Health Risk Factors for Seafood of Zhejiang Province, College of Food Science and Pharmacy, Zhejiang Ocean University, Zhoushan, China

**Keywords:** surimi, Bombay duck (*Harpadon nehereus*), gel properties, heating method, protein structure

## Abstract

This study investigates the effects of heating method, setting time, and setting temperature on the gel properties, water holding capacity (WHC), molecular forces, protein composition, protein conformation, and water transition of Bombay duck (BD) surimi gel. The obtained results demonstrate that the best gel properties are obtained by two-step heating at 30°C for 120 min while the hardness was 10.418 N and the breaking force was 4.52 N. Gel softening occurs at setting temperatures greater than 40°C due to the effect of endogenous enzymes in destroying the protein structure and increasing the hydrophobic and disulfide interactions. Low-field nuclear magnetic resonance spectra confirm that high two-step setting temperatures induce gel softening and the destruction of the surimi gel structure, as evidenced by the increased water migration at these temperatures. Of all protein conformations in the gel, the β-sheet structure, decreases from 38.40% at 30°C to 11.75% at 60°C when the setting time is 60 min, is the most susceptible to gel softening. Overall, the data reported herein provide a scientific basis for the development of new BD surimi products on an industrial level.

## Graphical Abstract

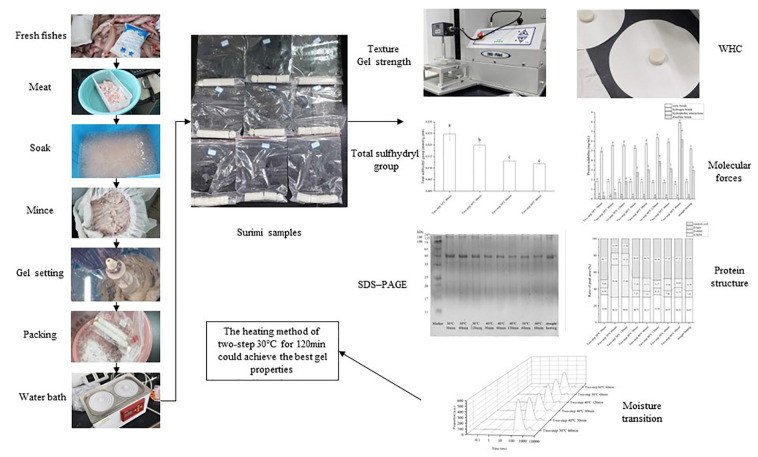

## Introduction

Surimi, a gelatinous food containing high concentrations of myofibrillar protein, is famous for its high nutritional value and diverse essential amino acid content. Surimi-based products are popular traditional foods in East Asia, as they have favorable taste and are easy to make. In general, surimi is prepared by gelation of salt-soluble myofibrillar proteins, and its properties depend on the gel network formed during heating. Therefore, the heating methods and strategies adopted during gelation may affect the properties and quality of the surimi gel (1). For instance, heating at temperatures less than 40°C results in the formation of a dense surimi gel network with enhanced properties, while heating at temperatures between 40 and 60°C results in the destruction or softening of the gel due to peptide bond breaking by endogenous enzymes such as serine and cysteine proteinases. The process occurring at T < 40°C is referred to as “Suwari (gel setting)” (2), whereas that taking place at 40 < T < 60°C is “Modori (gel softening)” (3, 4). Compared to straight heating strategy, the two-step heating strategy consisting of an extended Suwari step, followed by a short Modori step, effectively enhances the properties of the gel (5). Although the methods of surimi production have been thoroughly investigated, further research is needed to determine the optimum heating strategy including setting temperature and setting time of different species characterized by varying properties and muscle protein compositions. Such research has been conducted on Atlantic Croaker and Amur sturgeon (6, 7), but it must be extended to other species.

The main species used to prepare surimi products on the industrial level are Alaska Pollock (*Theragra chalcogramma*) and Pacific Whiting (*Merluccius productus*) (8). Considering that the yields of these species are significantly affected by climate change and global warming (9), it is imperative to seek alternative species such as Bombay duck (*Harpadon nehereus*) (BD). As one of the main aquatic products in East China Sea, the easily captured BD species with tender texture is quite popular (10). However, the high moisture content of this species leads to its rapid deterioration, which impedes its transport and storage, as well as reduces its shelf-life (11). To overcome this limitation, BD is made into surimi products. The use of this species in surimi production provides an alternative to the declining traditional strategies and enriches the product types in the market. Thus far, the protein gel properties and optimum gelation strategy for BD remain unknown. However, this information is needed to industrialize BD surimi in the future. In this study, we explore the heat-induced changes in BD protein molecule crosslinking under different conditions to determine the optimum heating strategy that yields the best gel properties of BD surimi.

## Materials and methods

### Materials and chemicals

Bombay duck specimens were caught in Zhoushan, East China Sea, during the month of September. The specimens were transferred to a foam box containing ice and then were transported to the lab within 6 h of catching. Subsequently, the fish were decapitated, gutted, skinned, and deboned before being minced by an FP3010 food processer (Braun, German) operated on low level. To ensure freshness, all sample preparation steps were carried out at 4°C. All chemicals (analytical grade) were purchased from Sinopharm Chemical Reagent Co., Ltd. (Shanghai, China).

### Preparation of surimi samples

Surimi samples were prepared according to the method of Gao et al. (12), with slight modifications. The BD mince was soaked with water (4°C) at the mince/water ratio of 1:10 (w/w) for 60 min. After squeezing and draining by gauze, the mince which had 85.71% moisture content was completely broken up using an FP3010 food processer (Braun, German); then, it was well-distributed in 3% sodium chloride for 10 min. The temperature was maintained between 0 and 4°C throughout the entire process. Subsequently, the mince was filled in plastic casings with a folding diameter of 36 mm, followed by heating in an HHS-11-2 constant-temperature water bath (BOXUN, China) under different conditions: (A) straight heating at 90°C for 30 min; (B) two-step heating; 3 groups setting at 30°C for 30, 60, and 120 min separately and then heating at 90°C for 30 min; 3 groups setting at 40°C for 30, 60, and 120 min separately and then heating at 90°C for 30 min; and 2 groups setting at 50°C and 60°C for 60 min, respectively, and then heating at 90°C for 30 min.

### Texture profile and gel strength analyses

The gel properties were analyzed according to the method of Nyaisaba et al. (13), with slight modifications. To determine the texture profile and gel strength, 20-mm cylindrical surimi samples were cut. The texture and gel strength of the heated surimi was analyzed using a PILOT texture analyzer (Food Technology Corporation, USA) equipped with a 250 N force sensing element. The trigger force was set as 0.6 N, the BD surimi samples were pressed to 30% of its initial thickness at a constant speed of 1 mm/s for texture analyzing; and for gel strength, the BD surimi samples were pressed to 10-mm at a constant speed of 1 mm/s. A 50-mm-diameter cylindrical probe was used to determine the hardness, springiness, gumminess, and chewiness of the samples, while a 5-mm spherical probe was used to determine gel strength.

### Determination of water holding capacity

The water holding capacity (WHC) of surimi samples, i.e., the weight of water retained in these samples, was determined according to the method of Nyaisaba et al. (13), with some modifications. Three-gram pieces of surimi (∼5 mm) were accurately weighed on an analytical balance (M_1_), wrapped tightly in filter paper, then centrifuged in tubes with cotton on the bottom. After six min of centrifugation at 4000 × *g*, the surimi samples were weighed again (M_2_). The WHC was determined as the percent ratio of M_2_ to M_1_.


(1) $$W\,H\,C=\frac{M_{2}}{M_{1}}\times 100\%$$

### Assessment of molecular forces

The solubility of proteins in the gel was assessed based on the method proposed by Niu et al. and Chu et al. (14, 15), with some modifications. Four chemical reagents [0.6 mol/L NaCl (SA); 0.6 mol/L NaCl, 1.5 mol/L urea (SB); 0.6 mol/L NaCl, 8 mol/L urea (SC); and 0.6 mol/L NaCl, 8 mol/L urea, 0.5 mol/L β-mercaptoethanol (SD)] were used to cleave interactions, dissolve the surimi protein, and examine ionic bonds (SA), hydrogen bonds (SB minus SA), hydrophobic interactions (SC minus SB), and disulfide bonds (SD minus SC) in the gel. For this purpose, 10 mL of each prepared reagent were mixed with 1.0 g of surimi sample and homogenized using a D-160 homogenizer (DLAB, China) operated at 8000 r/min for 1 min. The homogenates were kept at 4°C for ∼60 min, then they were centrifuged at 8,000 × *g* for 15 min using an X1R centrifuge (Thermo, Germany). The molecular forces were assessed based on the protein content of the supernatant, and the Coomassie brilliant blue method (16) was applied to account for the effect of β-mercaptoethanol on color development. The obtained results are expressed in mg soluble protein/mL of homogenate.

### Determination of total sulfhydryl group

The total mercapto (–SH) measurement kit (Jiancheng, China) was used to determine the concentration of total sulfhydryl group in the protein, according to the manufacturer’s instructions. Each surimi sample was added to Tris-HCl buffer [8 M urea, 2% sodium dodecyl sulfate (SDS), 10 mM EDTA, pH 8.0] and bathed with dithio-bis-(2-nitrobenzoic acid) (DTNB), a compound that reacts with the sulfhydryl group to form a yellow product. After 15 min of reaction at 37°C, the absorbance of the yellow product was measured at 412 nm using a U-2800 UV-Vis spectrophotometer (HITACHI, Japan). The concentration of sulfhydryl was calculated based on the measured absorbance value and the absorption coefficient of 13,600 M^–1^ cm^–1^. The calculated concentration values are expressed in mmol total SH/g of protein.

### SDS–polyacrylamide gel electrophoresis

SDS–PAGE analysis was used to determine the protein patterns of surimi gels according to the method of Cao et al. (17), with few modifications. The samples were prepared by mixing 27 mL of 5% (w/v) SDS solution with 3 g of the surimi gel then homogenizing the mixture in a D-160 homogenizer (DLAB, China) operated at 10000 rpm for 2 min. Subsequently, the homogenates were incubated at 85°C for 60 min to completely dissolve the proteins. After centrifugation (X1R centrifuge; Thermo, Germany) of the prepared samples at 8000 × *g* and 4°C for 20 min, the concentration of the protein in the supernatant was measured based on the Coomassie brilliant blue method. Thereafter, each sample was mixed with the sample buffer (0.5 M Tris–HCl solution containing 4% SDS, 20% glycerol, and 10% β-ME; pH 6.8) at the sample/sample buffer ratio of 1:3 (v/v) and boiled for 4 min. The boiled mixtures (15 μg protein) were loaded onto polyacrylamide gels (5% stacking gel and 15% running gel) along with the PR1920 protein standard marker (Solarbio, China), and electrophoresis was carried out at 80 V then 120 V using an EPS-300 unit (Tanon, China). After electrophoresis, the gel was stained with staining solution (0.02% Coomassie Blue R-250, 50% methanol, 7.5% acetic acid) then destained with destaining solution (50% methanol, 7.5% acetic acid).

### Determination of protein structure

The protein structure of the BD surimi gel was analyzed by Raman spectroscopy (DXR2xi Raman spectrometer; Thermo Fisher Scientific, USA), according to the method of Herrero et al. (18). The samples were scanned three times in the range of 50–3400 cm^–1^, using a λ = 785 nm semiconductor laser operated at room temperature. After smoothing and baseline correction, the spectral peaks were differentiated and imitated using the Peakfit software version 4.1.2 (Systat Software, USA). The intensities of the Raman signals were normalized relative to the phenylalanine peak at 1003 cm^–1^, as it is insensitive to the microenvironment.

### Determination of moisture transition

The moisture transition of the BD surimi gel was analyzed *via* low-field nuclear magnetic resonance (LF-NMR), according to the method of Ma et al. (19). Relaxation measurements were conducted on cylindrical gel samples (∼20 mm) using a 12.8 MHz Macro MR LF-NMR analyzer (Niumag, China) operated at room temperature. The T_2_ relaxation time was determined based on the Carr-Purcell Meiboom-Gill (CPMG) sequence at SW (kHz) = 100; RFD (ms) = 0.200; O_1_ (Hz) = 17244.04; RG_1_ (db) = 20.0; *P*_1_ (μs) = 7.52; DRG_1_ = 3; TD = 220000; PRG = 1; TW (ms) = 1500.000; *P*_2_ = 13.52; NS = 8, TE (ms) = 0.220; and NECH = 10000.

### Statistical analysis

All experiments were performed in triplicate at least, and the data are presented as mean ± standard deviation. The IBM SPSS statistics 26 software (SPSS Institute Inc., USA) and OriginPro 2021 (OriginLab Corporation, USA) were used for statistical analysis, and *P* < 0.05 was considered to indicate a significant difference between means (Duncan’s multiple range testing).

## Results and discussion

### Physical properties of surimi gel

**FIGURE 1:**
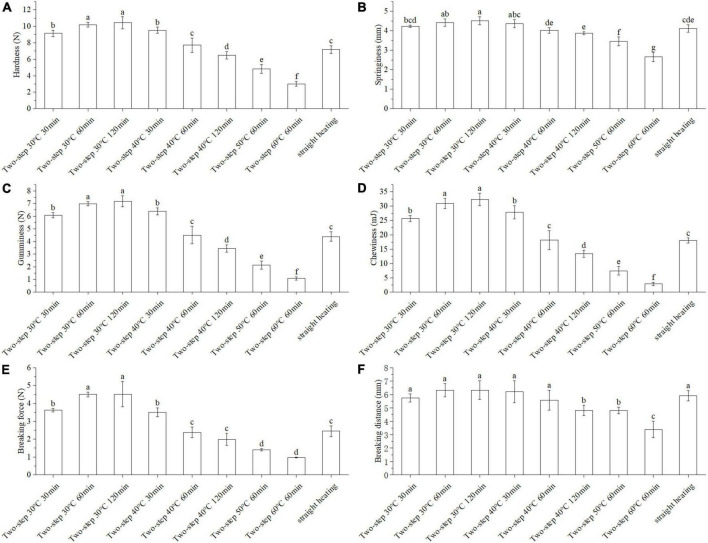
Changes of texture and gel strength under different heating conditions. Different letters showed the significant changes (*p* < 0.05). **(A)** Hardness, **(B)** springiness, **(C)** gumminess, **(D)** chewiness, **(E)** breaking force, and **(F)** breaking distance. Data were obtained as the mean ± standard deviation (*n* = 5).

Texture and gel strength were important gelation properties that could be used to assess the degree of gel network formation *via* protein crosslinking (20). As shown in Figure 1A, hardness decreased significantly with increasing setting temperature (two-step heating strategy) at constant setting time (60 min). Based on the available literature, the surimi gel could form a network structure with gel setting at 30°C; however, when the temperature reached 40°C, gel softening occurred, and endogenous enzymes in the surimi of BD, such as serine and cysteine proteinases, started to act (3, 4). The gel softening induced by heating temperatures between 50 and 60°C further deteriorated the properties of the gel (21). Herein, we showed that by applying the two-step setting time strategy, different hardness trends may be obtained at different setting temperatures. Indeed, hardness increased with increasing setting time at 30°C, but it decreased with increasing setting time at 40°C due to gel softening. At 30°C, the disintegration and rearrangement of actin, troponin, tropomyosin, and light chain meromyosin and the protein cross-linking by hydrophobic forces and disulfide bonds formed a dense gel network (15, 22). Not only that, endogenous transglutaminase in BD could promote the cross-linking of glutamine and lysine reactions in myofibrillar proteins [epsilon − (γ-glutamyl) -Lysine cross-linkage] creating a more stable gel network (23). When the temperature was increased to 40°C, gel softening took place, resulting in the gradual destruction of the gel structure (24). Similar results were reported by Gao et al. for Amur Sturgeon surimi (6). Springiness (Figure 1B), gumminess (Figure 1C), chewiness (Figure 1D), and gel strength (Figures 1E,F) exhibited the same trend as hardness.

### Water holding capacity

**FIGURE 2:**
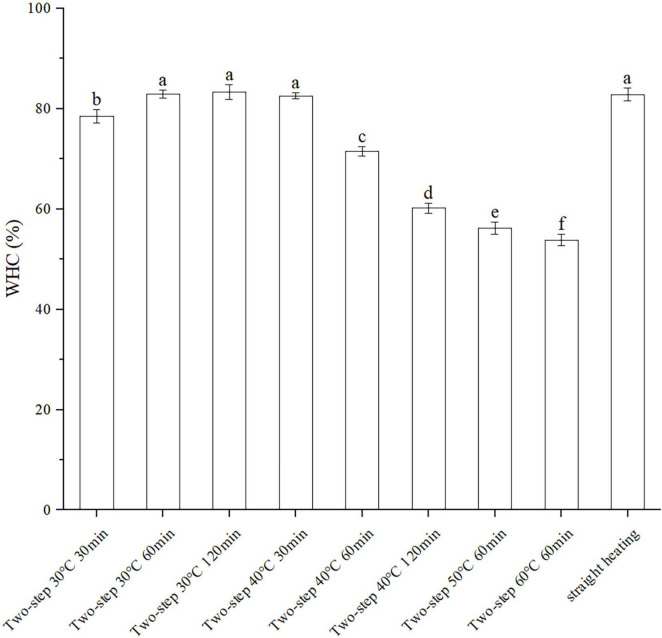
Changes of WHC under different heating conditions. Different letters showed the significant changes (*p* < 0.05). Data were obtained as the mean ± standard deviation (*n* = 5).

The WHC reflected the ability of surimi gels to keep water under pressing conditions and was directly related to the density of the surimi gel network structure. As shown in Figure 2, the WHC of the BD surimi gel was increased when lower setting temperature for two-step heating was employed. Moreover, the effect of two-step setting time on WHC varied at different setting temperatures. At 30°C, the WHC increased with increasing setting time; however, it decreased with time at 40°C, similar to other gel properties. Compared to the straight heating method, better WHC was obtained with two-step heating. Therefore, the gel setting and gel softening phenomena occurring during the process of two-step heating may affect the strength of the gel network (4, 6).

### Molecular forces

**FIGURE 3:**
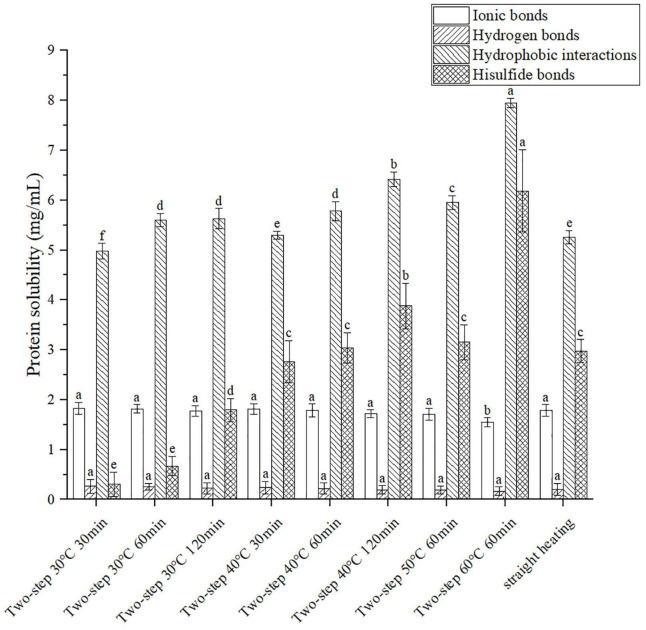
Changes of molecular forces under different heating conditions. Different letters showed the significant changes (*p* < 0.05). Data were obtained as the mean ± standard deviation (*n* = 5).

The molecular forces in the gel, including ionic bonds, hydrogen bonds, hydrophobic interactions, and disulfide bonds, were assessed using different chemical reagents to break different forces and dissolve the protein. The degree of gelation was determined based on the amount of dissolved protein (22). As shown in Figure 3, both ionic bonding and hydrogen bonding decreased slightly with increasing setting temperature of the two-step heating process; however, the decreasing trend was not obvious. This agreed well with the results reported by Liu et al. (25). Although the effect of ionic and hydrogen bonds on gel formation was less than that of hydrophobic interactions and disulfide bonds, all molecular forces contributed to the conformation stability of proteins (26). For example, hydrogen bonding was important for the stability of the β-sheet protein conformation, which was related to gel properties (27). Compared to ionic and hydrogen bonds, the content of hydrophobic interactions and disulfide bonds in the gel structure were greater, as reported by Xiong et al. (28). The results presented in Figure 3 demonstrated that hydrophobic interactions increased with increasing setting temperature and setting time, possibly due to the unfolding of the protein structure at higher temperatures at which gel softening occurred. The unfolding of the protein exposed the internal hydrophobic groups, resulting in increased hydrophobic interactions (25, 29). Moreover, at T ≥ 40°C, endogenous proteases destroyed the secondary and tertiary structures of the surimi protein (gel softening), resulting in the exposure of amino acid residues with hydrophobic groups (3, 4, 30). Disulfide bonds exhibited the same trend as hydrophobic forces. At 30°C setting temperature, the content of disulfide bonds increased with increasing setting time, even though the protein structure was not completely unfolded (31). As the temperature was increased above 40°C, the protein gradually unfolded and decomposed (gel softening), thereby exposing the inner sulfhydryl groups and promoting the formation of disulfide bonds (32). Interestingly, the temporal variation trends of hydrophobic and disulfide forces observed during gel setting (30°C) were similar to those of WHC and gel properties, while the trends recorded during gel softening (above 40°C) were opposite. Such difference in trends had also been reported by Yi et al. (33) for disulfide bonds and was primarily attributed to the opening of the peptide-chain by gel softening (34).

### Total sulfhydryl groups

**FIGURE 4:**
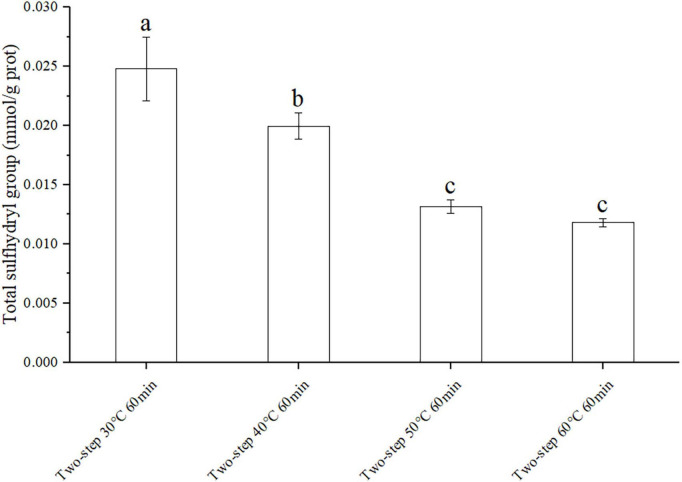
Changes of total sulfhydryl group under different heating conditions. Different letters showed the significant changes (*p* < 0.05). Data were obtained as the mean ± standard deviation (*n* = 5).

Knowing that disulfide bonds were formed by sulfhydryl groups, the effect of different degrees of gel softening on total disulfide bonds may be determined by measuring the content of total sulfhydryl groups (35). The experimental groups with large gradient difference of gel softening were selected such as different setting temperatures to prove the origin of massive disulfide bond formation. The results in Figure 4 demonstrated that the content of total sulfhydryl decreased with increasing setting temperature due to gel softening (32). At 30°C, gel softening did not occur, and the internal structure of the protein remained intact. Therefore, only few sulfhydryl groups were exposed and could form disulfide bonds at this condition. Meanwhile, at 40°C, the protein structure was destroyed by endogenous proteases, resulting in the formation of numerous disulfide bonds due to the exposure of internal sulfhydryl groups (36, 37).

### SDS–polyacrylamide gel electrophoresis

**FIGURE 5:**
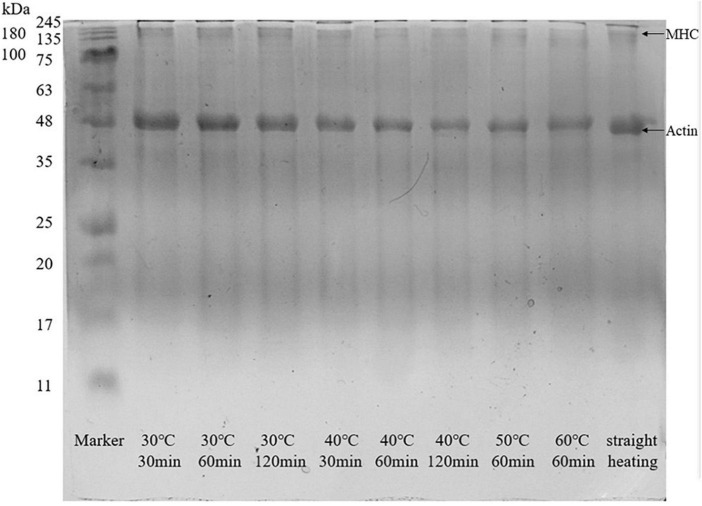
SDS-PAGE of surimi gel under different heating conditions.

As demonstrated by Du et al. (38), myosin heavy chain (MHC, 220 kDa) and actin (AC, 43 kDa) had a positive effect on the formation of surimi gel. Based on the SDS-PAGE results illustrated in Figure 5, MHC and AC were clearly observed in the protein banding patterns of gels. The bands of MHC in all groups were shallow, which could be attributed to the low content of endogenous transglutaminase in surimi and the limited degree of promoting the cross-linking of glutamine and lysine reactions [epsilon − (γ-glutamyl) -Lysine cross-linkage] in myofibrillar proteins (23). Meanwhile, the content of disulfide bonds also supported this phenomenon; in Figure 3, the content of disulfide bond that facilitated protein cross-linking was low at 30°C; when setting temperature >40°C, gel softening caused the break of peptide chain and the newly generated disulfide bond only linked small protein fragments. Besides, in two-step heating processes, setting time had little effect on MHC and AC bands at the setting temperature of 30°C. However, at T ≥ 40°C, the MHC bands became shallower and wider, and the content of MHC decreased with increasing setting temperature and time, especially at 50 and 60°C. This effect of gel softening on the SDS-PAGE of surimi gel had also been reported by Gao et al. (12), and it may be attributed to the breakdown of MHC by endogenous proteases such as serine and cysteine proteinases (39). Previously, Hu, Morioka and Itoh (40) had shown that cysteine protease cathepsin L affected gel properties by influencing MHC. Like MHC, the AC bands became shallower, and the content of AC in gels decreased at higher setting temperatures and longer times. Such a decrease may also be related to endogenous proteases that acted on AC and leaded to enzymolysis. Indeed, studies showed that the cathepsin L enzyme isolated from the muscle of mature chum salmon played an active role in the hydrolysis of major protein components of myofibril, such as connectin, nebulin, myosin, a-actinin, and troponins (36, 41). In short, the gel softening phenomenon induced by high setting temperatures resulted in the destruction of MHC and AC, which made the bands shallower and wider. This was consistent with the gel properties and WHC trends discussed above.

### Protein structure

**FIGURE 6:**
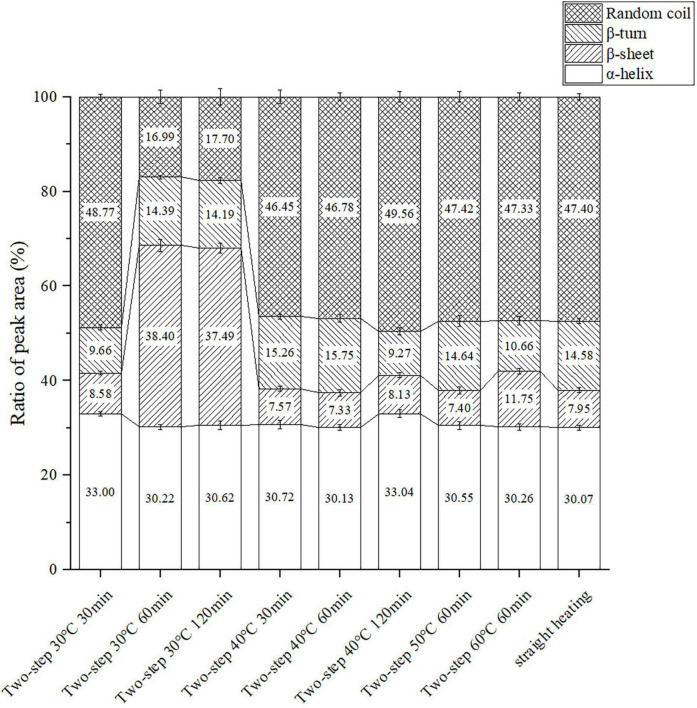
Changes of the protein secondary structure content under different heating conditions. Data were obtained as the mean ± standard deviation (*n* = 3).

Changes in protein conformation during heating may be used to assess the protein gel structure at the microscopic level. The proportion of α-helix, β-sheet, β-turn, and random coil conformations could be obtained by differentiating and imitating the Raman peaks in the amide I region (42). As shown in Figure 6, variations in setting temperature during two-step heating at 60 min setting time induced changes in the protein secondary structure. The setting temperature did not affect the rate of α-helix extension and decomposition, and the proportion of the α-helix conformation remained almost the same at different temperatures. Meanwhile, the proportion of combined β-sheet (43, 44) and β-turn (33, 45) structures, which had positive effects on gel network formation and gelation properties, gradually decreased with increasing setting temperature, and the proportion of random coil structures, which were associated with diminished gel properties (46), increased. In addition, the data in Figure 6 showed that the β-sheet structure was more susceptible to gel softening at higher setting temperatures than the β-turn counterpart. When the temperature changed from 30 to 40°C, the proportion of β-sheet decreased sharply (the proportion of β-turn increased slightly), which indicated that the rate of β-sheet extension and decomposition was extremely rapid at conditions of low degree gel softening. Contrarily, when the temperature was increased from 50 to 60°C, rapid changes in the rate of β-turn extension and decomposition occurred, resulting in a sharp decrease in the proportion of this conformation (the proportion of β- sheet increased slightly). Such opposite trends may be explained by the destruction of hydrogen bonds in β-sheet structures during gel softening. The β-sheet structure was maintained by hydrogen bonding, and a decrease in the proportion of this structure signified a decrease in hydrogen bonding interactions, as well as in the stability of secondary and tertiary protein structures (27).

Different effects of setting time on the proportion of protein secondary structure were observed at different setting temperatures. At 30°C, the proportion of β-sheet and β-turn structures significantly increased, while that of the α-helix structure decreased at 60 min compared to 30 min. This confirmed that gel setting and the transformation of protein secondary structure to β-sheet and β-turn were positively correlated (43, 45), and that the decrease in α-helix proportion contributed to the formation of a stable gel structure (5). When the setting time was increased to 120 min, the β-sheet + β-turn proportion slightly decreased due to the instability and slight hydrolysis of the hydrogen bond subjected to long-term heating in a water bath. At 40°C setting temperature, gel softening began and the gel network was gradually destroyed by endogenous enzymes, resulting in downgraded gel properties. All data (including those obtained using the straight heating strategy) were consistent with the trends of gel properties and WHC. The results reflected the importance of the gel setting process for gel formation, particularly at the microscopic level.

### Moisture transition

**FIGURE 7:**
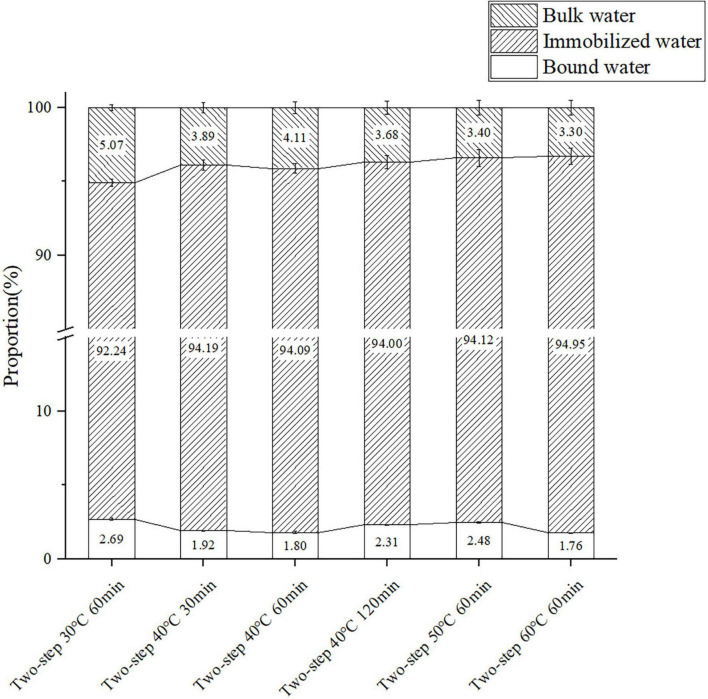
The content of the three components of surimi gels formed under different gel softening degree. Data were obtained as the mean ± standard deviation (*n* = 3).

**FIGURE 8:**
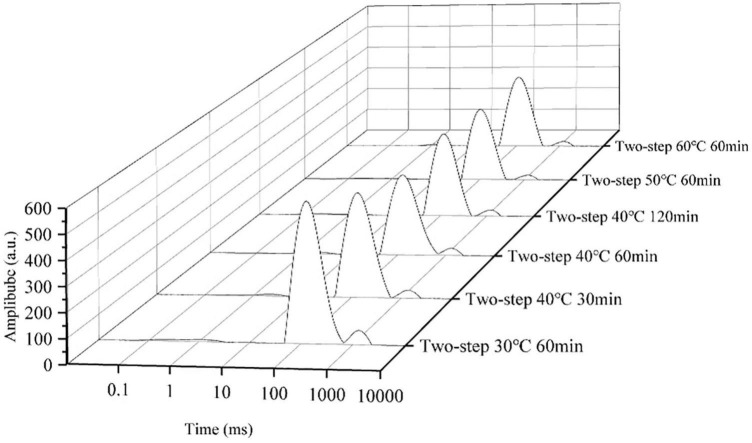
The distributions of T_2_ relaxation times of surimi gels formed under different gel softening degree.

The T_2_ relaxation time determined by LF-NMR characterized the water state in the surimi gel, and the three peaks in the LF-NMR spectrum represented bound water, immobilized water, and bulk water. The proportion of different forms of water, especially immobilized water, reflected the density of the gel network (47). Samples heated at 30°C for 60 min (no gel softening) were included in the control group, and samples heated at higher setting temperatures for different periods of time were included in test groups of varying gel softening degrees. The LF-NMR spectra presented in Figures 7, 8 demonstrated that the peak area of immobilized water rapidly declined at higher setting temperature and constant setting time (60 min) during two-step heating processes, while the proportion of immobilized water increased. Such variation may be attributed to the transition from immobilized water to bulk water and the massive loss of bulk water during heating. When gel softening occurred, the gel network structure was destroyed, and a large amount of immobilized water was transformed into bulk water, which oozed out of gradually enlarged pores, resulting in an increased proportion of immobilized water (48). Furthermore, at 40°C two-step setting temperature, gel softening became more obvious at longer setting times, and the proportion of immobilized water decreased. Meanwhile, for bulk water, the damage of gel structure caused by gel softening leaded to the direct leakage of a large amount of bulk water, which resulted in the decrease of the peak area and proportion of bulk water with the development of gel softening. In short, gel softening destroyed the gel network and accelerated the process of water migration (49).

## Conclusion

This study provides a comprehensive analysis of the heating strategy of BD surimi. The results show that gel softening does not occur at 30°C, and that the two-step heating method of 30°C for 120 min yields the best gelation properties. Due to the phenomenon of gel setting, the optimal method of two-step heating is better than that of straight heating. In addition, Raman spectroscopy analysis shows that the β-sheet structure associated with gel properties is more susceptible to gel softening than other protein secondary structures.

## Data availability statement

The raw data supporting the conclusions of this article will be made available by the authors, without undue reservation.

## Author contributions

ML contributed to the investigation, methodology, formal analysis, data curation procedures, and writing the original manuscript draft. JY, HB, and YC contributed to the investigation and data curation procedures. YG contributed to conceptualization, project administration, supervision, reviewing, and editing the draft. SD contributed to validation, reviewing, and editing the draft. All authors read and agreed to the published version of the manuscript.
